# New and Simple Approach for Preventing Postoperative Peritoneal Adhesions: Do not Touch the Peritoneum without Viscous Liquid—A Multivariate Analysis

**DOI:** 10.1155/2012/368924

**Published:** 2012-01-26

**Authors:** Erhan Aysan, Hasan Bektas, Feyzullah Ersoz, Serkan Sari, Arslan Kaygusuz, Gulben Erdem Huq

**Affiliations:** Department of General Surgery, Bezmialem Vakif University, 80700 Istanbol, Turkey

## Abstract

*Background*. Postoperative peritoneal adhesions (PPAs) are an unsolved and serious problem in abdominal surgery. *Method*. Viscous liquids of soybean oil, octyl methoxycinnamate, flax oil, aloe vera gel, and glycerol were used in five experiments, using the same methodology for each. Liquids were applied in the peritoneal cavity before and after mechanical peritoneal trauma. Results were evaluated by multivariate analysis. *Results*. Compared with the control group, macroscopic and microscopic adhesion values before (*P* < .001) and after (*P* < .05) application of viscous liquids significantly reduced PPAs. Values were significantly lower when liquids were applied before rather than after peritoneal trauma (*P* < .0001). *Discussion*. Viscous liquids injected into the peritoneal cavity before or after mechanical peritoneal trauma decrease PPA. Injection before trauma was more effective than after trauma. In surgical practice, PPA formation may be prevented or decreased by covering the peritoneal cavity with an appropriate viscous liquid before abdominal surgery.

## 1. Introduction

Postoperative peritoneal adhesions (PPAs) are a serious complication experienced by more than three-quarters of patients who undergo abdominal surgery. PPAs are the most common causes of intestinal obstruction, infertility, and abdominal and pelvic pains [1–3]. PPAs also represent a serious economic problem. For example, a multicenter study including all abdominal surgery units in Sweden found that the annual loss due only to small bowel obstruction was more than US$6 million [4]. Various materials and/or techniques have been investigated to prevent or treat PPAs, but to date, no effective solution has been identified.

PPAs are potentially preventable, and several agents that act as barriers between adjacent peritoneal surfaces have been evaluated for prophylaxis [5]. We, the authors, have as a team been for years working on viscous liquids for preventing PPAs. Viscous liquids studied were ricini, zingiber, daphne, orange, sesame, opium, jasmine, chamomile, hazelnut, eucalyptus, mint, myrtle, honey, and soybean oils, as well as octyl methoxycinnamate, flax seed oil, aloe vera gel, and glycerol. Only the last five have been shown to prevent PPAs [6–10]. In addition to determining the effects of these liquids on preexisting PPAs, we assessed their ability to prevent peritoneal trauma. We hypothesized that covering peritoneal surfaces with these viscous liquids may prevent PPA formation by preventing peritoneal trauma.

## 2. Methods

Experiments were performed between 2006 and 2010 at the Experimental Animal Production and Research Laboratory of Cerrahpasa Medical School, Istanbul University. All animal protocols were approved by the Animal Ethics Committee of Istanbul University, and experiments were performed in accordance with the regulations governing the care and use of laboratory animals outlined in the Declaration of Helsinki.

The viscous liquids used were soybean oil (Soya yagi, Arifoglu Co.), octyl methoxycinnamate (Vazelin sivi, Galenik Ecza Co.), flax seed oil (Keten yagi, Arifoglu Co.), aloe vera gel (Natural Aloe Vera Gel, Arifoglu Co.), and glycerol (Gliserin, Adora Kimya Co.) [6–10].

We used a total of 160 Wistar outbred female albino rats (mean weight, 190 ± 25 g; mean age, 6.5 months). According to the 3R rule (replacement, refinement, reduction) for experimental animal research, we constructed only one control group for all five experiments, thereby reducing the number of animals by forty.

### 2.1. Anesthesia Technique

Each rat was anesthetized with 40 mg/kg body weight intramuscular ketamine (Ketalar, Parke Davis and Co., Inc.).

### 2.2. Adhesion Model

**Figure 1:**
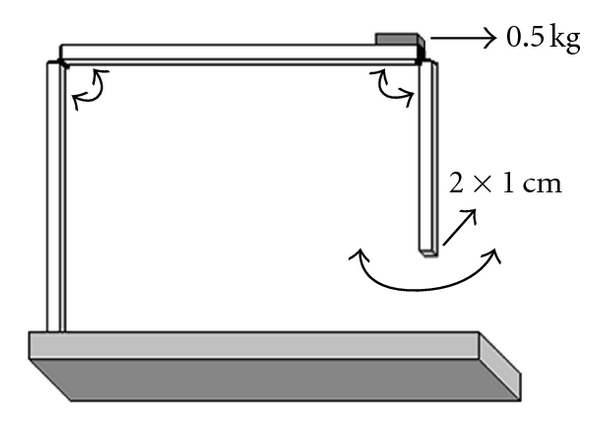
Standart peritoneal adhesion creation apparatus.

All adhesions were generated using a new apparatus of our own design (Figure 1). The set-up consists of a 20 × 10 cm surgical table with three arms: a stable vertical arm, a moving vertical arm, and a moving horizontal arm. The stable vertical arm works as a shaft and fixes the other two arms onto the surgical table. On the horizontal arm, a 0.5 kg weight is positioned most distal from the shaft. This weight corresponds to the pressure applied by a surgeon's fingertips on the intestinal surface of subjects via manipulation during laparotomies. The horizontal arm transmits the pressure effect of the weight to the lower part of the system by providing up-and-down movements of the system. The other moving arm, with a free pendulum moving along a vertical line, allows application of mechanical trauma (abrasion) on the peritoneal surface. The arm's surface in contact with the peritoneal area (2 × 1 cm) corresponds to the approximate area of a fingertip in contact with a surface (2 cm²). During the design of this model, a finger of a sterile, powder-free latex glove was placed onto this surface, thus sterilizing this area and increasing the human finger simulation. The anterior face of each rat's cecum was placed under the vertical moving arm; visceral peritoneal trauma was provided by pendulum movement of this arm ten times.

**Group 1 (*n* = 10)**

Control group. The adhesion model was generated.

**Group 2 (*n* = 50)**

In the absence of adhesions, intraperitoneal 0.1 mL of the following sterile viscous liquids was injected interperitoneally in five subgroups:

Group 2.1 (*n* = 10): soybean oil [6],
Group 2.2 (*n* = 10): octyl methoxycinnamate [7],
Group 2.3 (*n* = 10): flax seed oil [8],
Group 2.4 (*n* = 10): aloe vera gel [9],
Group 2.5 (*n* = 10): glycerol [10].

**Group 3 (*n* = 50)**

Prior to the generation of the adhesion model, 0.1 mL sterile viscous liquids was applied to cecum surfaces in five subgroups:

Group 3.1 (*n* = 10): soybean oil [6],
Group 3.2 (*n* = 10): octyl methoxycinnamate [7],
Group 3.3 (*n* = 10): flax seed oil [8],
Group 3.4 (*n* = 10): aloe vera gel [9],
Group 3.5 (*n* = 10): glycerol [10].

**Group 4 (*n* = 50)**

After the adhesion model was generated, cecum surfaces of five subgroups were covered with 0.1 mL sterile viscous liquids:

Group 4.1 (*n* = 10): soybean oil [6],
Group 4.2 (*n* = 10): octyl methoxycinnamate [7],
Group 4.3 (*n* = 10): flax seed oil [8],
Group 4.4 (*n* = 10): aloe vera gel [9],
Group 4.5 (*n* = 10): glycerol [10].

Ten days later, the rats were sacrificed by an overdose of intraperitoneal sodium pentothal (Pentothal Sodium Ampul, Abbott Co.). Laparotomy consisted of a reverse U-shape incision. Without damaging the formed adhesions, the anterior abdomen wall flap was retracted caudally, and adhesions observed in the peritoneal cavity were graded [11] macroscopically according to size and severity (Table 1).

Resected areas of adhesions were fixed in formol and, after dehydration, embedded in paraffin. Cross-sections of 5 mm thickness were prepared, stained with hematoxylin and eosin, and evaluated by light microscopy at a magnification of ×100. All evaluations were performed according to the microscopic fibrosis grading system [11] by a pathologist blinded to methods and groups (Table 2).

The primary outcome measure was a macroscopic adhesion score (average value of adhesion severity and adhesion size grade). The secondary outcome measure was a microscopic fibrosis grade.

### 2.3. Statistical Evaluation

All statistical evaluations were performed using the NCSS 2007 program for Windows. Besides standard descriptive statistical calculations (mean and standard deviation), the Kruskal-Wallis test was used to compare groups, and the post hoc Dunn's multiple-comparison test was used to compare subgroups. Statistical significance was defined as *P* < .05.

## 3. Results

The mean macroscopic adhesion score of the control group (Group 1) was 2.9 ± 0.42 and the mean microscopic adhesion score of this group was 2.8 ± 0.22. Scores of all rats receiving injections of viscous liquids in the absence of adhesions (Group 2) were 0. Rats receiving injections of viscous liquids prior to the generation of adhesions (Group 3) had macroscopic adhesion scores significantly lower than those of the control group (0.31 ± 0.49, *P* < .001, Table 3). Moreover, the mean macroscopic adhesion scores of rats injected with viscous liquids following to the generation of adhesions (Group 4) were lower than those of the control group (1.45 ± 1.29, *P* < .05, Table 4). Group 3 had significantly lower mean macroscopic adhesion scores than Group 4 (*P* < .0001).

Overall, the mean microscopic adhesion scores of Group 3 (0.50 ± 0.54, *P* < .001, Table 5) and Group 4 (1.70 ± 0.75, *P* < .05, Table 6) differed significantly from those of the control group (2.80 ± 0.22). Group 3 also had significantly lower mean microscopic adhesion scores than Group 4 (*P* < .0001).

## 4. Discussion

Since 1999, we have focused on the use of viscous liquids to prevent PPAs and have tested numerous kinds. Our first international manuscript (2002) is related to honey [12]. Because PPAs result from peritoneal trauma [1, 2], two fundamental methods are used to prevent PPA-related complications: the prevention of adhesion formation, and the treatment of adhesions after they formed. The second method is particularly complex because it relates to the wound healing process. This process involves multiple cell populations, the extracellular matrix and the action of soluble mediators such as growth factors, and cytokines, with some steps and molecular actors still unclear [13]. Because of this complexity, we focused on preventing PPAs by covering the peritoneal surface with various viscous liquids that do not negatively affect vital tissues, especially peritoneal mesothelial cells. Covering peritoneal surfaces with viscous liquids may prevent the peritoneum from experiencing mechanical trauma, both by preventing direct contact with the trauma-inducing material and by dispersing any focused pressure via fluid surface tension. We tested our hypothesis using five viscous liquids: soybean oil, octyl methoxycinnamate, flax seed oil, aloe vera gel, and glycerol.

We designed and utilized for the first time the apparatus used to create abrasions in the present study. By means of this apparatus, we were able to standardize each component of peritoneal trauma. The surface area of trauma was standardized by fixing the size of the surface that would come in contact with the peritoneum (2 × 1 cm). Trauma location was standardized by making the apparatus fixed and stable so that the pendular movement always affected the same point in each subject, thereby creating abrasions at the same location. The number of incidents of trauma was standardized by using the same number of pendular movements, and pressure was standardized by using a standard force derived from a 500 g weight.

In this extended researches period, we had two major aims: (1) to identify one or more liquids whose specific properties could prevent PPA, and (2) to test our hypothesis that PPAs could be prevented by prevention of peritoneal trauma via covering peritoneal surfaces with approprite viscous liquids. We found that soybean oil, octyl methoxycinnamate, flax seed oil, aloe vera gel, and glycerol were effective in preventing PPAs, with glycerol and flax seed oil being the most effective. Multivariate analysis showed that application of these liquids before or after the generation of peritoneal trauma significantly decreased PPAs. Moreover, application before trauma was significantly more effective than application after trauma.

Results of macroscopic evaluation relate to the clinical effectiveness of this new approach. Results of microscopic evaluation either support the macroscopic results or explain the mechanism of PPAs. In order to prevent peritoneal trauma directly (mechanically), histopathologic fibrosis values of the before-application groups were statistically differently diminished than those of control and after-application groups.

In conclusion, we have shown that application of viscous liquids to the peritoneal cavity before or after mechanical peritoneal trauma decreased PPA formation, with application before trauma being more effective. It is important to note that our findings result from experiments conducted in animals and need to be evaluated in humans. It is important point that our results are conducted in animals and need to be evaluated in humans. Application of a viscous liquid before peritoneal trauma is the new, simple and also cost-effective approach to preventing of PPA formation. Thus, in surgical practice, PPA formation may be prevented or decreased by applying an appropriate viscous liquid to the entire intraperitoneal cavity or area of manipulation before laparotomy/laparoscopy. Future studies on the use of viscous liquids with no negative effects on vital tissues, especially peritoneal mesothelial cells with different application modalities, are needed for more reliable results.

## Figures and Tables

**Table 1:** Definitions of macroscopic peritoneal adhesion grading system according to size and severity [11].

Grades	Adhesion size	Adhesion severity
0	No adhesion	No adhesion
1	Presence of adhesion in 25% of the area	Spontaneously separating adhesion
2	Presence of adhesion in 50% of the area	Separation of adhesion with traction
3	Whole area covered with adhesion	Separation of adhesion with a sharp dissection

**Table 2:** Definitions of microscopic fibrosis grading system [11].

Grades	Definition
0	No fibrosis (no fibroblasts and/or collagen fibers)
1	Slight fibrosis (few fibroblasts and/or collagen fibers)
2	Median fibrosis (more fibroblasts and/or collagen fibers)
3	Severe fibrosis (lots of fibroblasts and/or collagen fibers)

**Table 3:** Macroscopic adhesion scores of subgroups 3 (score of control group: 2.90 ± 0.21).

	Prior the generation of adhesions	Difference with control groups
Glycerol	0.00 ± 0.00	*P* < .001
Octyl methoxy	0.40 ± 0.84	*P* < .001
Soy bean oil	0.50 ± 0.71	*P* < .001
Flax oil	0.10 ± 0.32	*P* < .001
Aloe vera gelly	0.55 ± 0.60	*P* < .001

*Total*	0.31 ± 0.49	*P* < .001

**Table 4:** Macroscopic adhesion scores of subgroups 4 (score of control group: 2.90 ± 0.21).

	After the generation of adhesions	Difference with control groups
Glycerol	0.30 ± 0.95	*P* < .001
Octyl methoxy	1.80 ± 2.39	*P* < .05
Soy bean oil	1.90 ± 0.94	*P* < .05
Flax oil	0.65 ± 1.80	*P* < .01
Aloe vera gelly	2.60 ± 0.39	*P* > .05

*Total*	1.45 ± 1.29	*P* < .05

**Table 5:** Microscopic adhesion values of subgroups 3 (value of control group: 2.80 ± 0.42).

	Prior the generation of adhesions	Difference With control groups
Glycerol	0.60 ± 0.51	*P* < .01
Octyl methoxy.	1.30 ± 0.48	*P* < .05
Soy bean oil	0.20 ± 0.42	*P* < .001
Flax oil	0.20 ± 0.42	*P* < .001
Aloe vera gelly	1.20 ± 0.91	*P* < .05

*Total*	0.50 ± 0.54	*P* < .001

**Table 6:** Microscopic adhesion values of subgroups 4 (value of control group: 2.80 ± 0.42).

	After the generation of adhesions	Difference with control groups
Glycerol	1.60 ± 0.70	*P* < .01
Octyl mMethoxy.	2.40 ± 0.70	*P* > .05
Soy bean oil	1.50 ± 1.35	*P* < .05
Flax oil	0.40 ± 0.52	*P* < .001
Aloe vera gelly	2.60 ± 0.51	*P* > .05

*Total*	1.70 ± 0.75	*P* < .05
